# Simultaneous Cervical and Lumbar Vertebral Fracture-Dislocation in an Ankylosing Spondylitis Patient: A Case Report

**DOI:** 10.7759/cureus.85462

**Published:** 2025-06-06

**Authors:** Muhammad Farhan Md Yusoff, Amir Fariz Zakaria, Kean Loong Ong, Asrul Syamin Hisyam Yong, Faiq Abdulrahman

**Affiliations:** 1 Department of Orthopedics and Traumatology, Hospital Sungai Buloh, Sungai Buloh, MYS; 2 Department of Orthopedics and Traumatology, Hospital Universiti Kebangsaan Malaysia, Kuala Lumpur, MYS; 3 Spine Unit, Department of Orthopedics, Hospital Sungai Buloh, Sungai Buloh, MYS

**Keywords:** ankylosing spondylitis, burst fracture, cement augmentation, neurological deficit, vertebral fusion

## Abstract

Fractures in ankylosing spondylitis (AS) patients tend to occur due to the absence of motion between vertebrae, poor bone quality, and a long lever arm that generates extension force. However, most patients have a history of at least minor trauma. Due to the spine's extreme instability in AS patients, even neurologically intact individuals can experience secondary neurological deterioration after unprotected transfers or manipulations.

We report a 59-year-old gentleman presenting late with acute neurological symptoms following a history of trauma. Fracture diagnosis in patients with ankylosing spondylitis (AS) poses a significant challenge due to vertebral fusion and altered spinal architecture. In this case, the patient sustained simultaneous burst fractures at C6 and L4 levels, with a significant neurological deficit. He underwent posterior spinal instrumentation and fusion (PSIF) from L1 to S2, PSIF from C3 to T2 with bone cement augmentation, and L4/L5 decompression laminectomy. Following a thorough investigation and appropriate surgical management, the patient was discharged home postoperatively without any further neurological deterioration.

## Introduction

Ankylosing spondylitis (AS) is a chronic inflammatory spondyloarthropathy marked by sacroiliitis and lesions in the axial joints. The degeneration of these joints results in paravertebral benign ossification and ankylosis, ultimately leading to the formation of a "bamboo spine" [1]. A consequence of this extensive spinal ossification is sagittal deformity, including thoracic kyphosis and reduced flexibility. As AS progresses, fractures or dislocations can occur even with minimal or no force due to increased vertebral osteoporosis and bone brittleness. These fractures-dislocations, which occur at the cervical level in 81% of patients, act similarly to long bone fractures, with the long lever arm being highly unstable. The development of neurological deficits is a recognized and serious complication following fracture-dislocation in AS patients, making reliable external fixation crucial both before and during surgery [2].

## Case presentation

A 59-year-old Chinese gentleman presented with complaints of neck pain following a motor vehicle accident. Examination and imaging reveal normal findings. He was put on a cervical immobilizer.

Three weeks later, he returned to the emergency department, reporting bilateral lower limb weakness and difficulty walking. There were no recent falls, and he denied any urinary or bowel incontinence. Neurological examination revealed intact function in the upper limbs from C5 to T1 bilaterally. However, there was a complete loss of power below L4 bilaterally (Medical Research Council {MRC}: 0), with preserved sensation.

**Figure 1:**
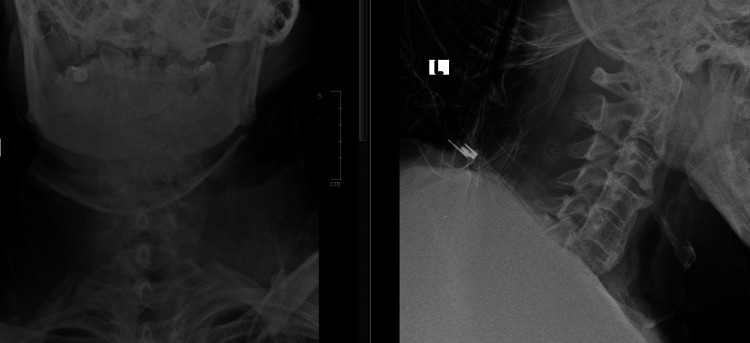
Anteroposterior and lateral view of plain cervical radiograph: no obvious fracture visible

**Figure 2:**
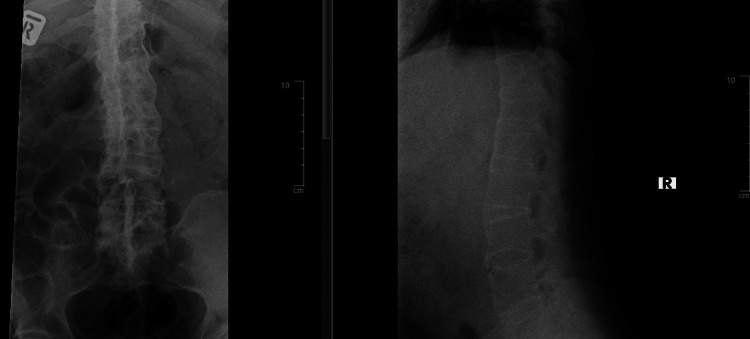
Plain lumbosacral radiograph: no visible fracture with "bamboo spine"

Plain cervical radiograph (Figure 1) and lumbosacral radiograph (Figure 2) show a typical "bamboo spine" with no obvious fracture or osteolysis. A repeated CT scan showed a C6 body fracture with a chance fracture of L4 and a chip fracture of L3 vertebrae. The fractures in the spine and sacroiliac joints suggest ankylosing spondylitis.

**Figure 3:**
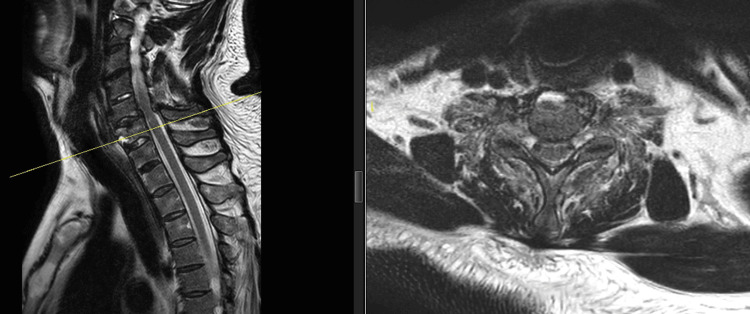
T2-weighted image shows C6 body fracture with spinal stenosis and cord edema

**Figure 4:**
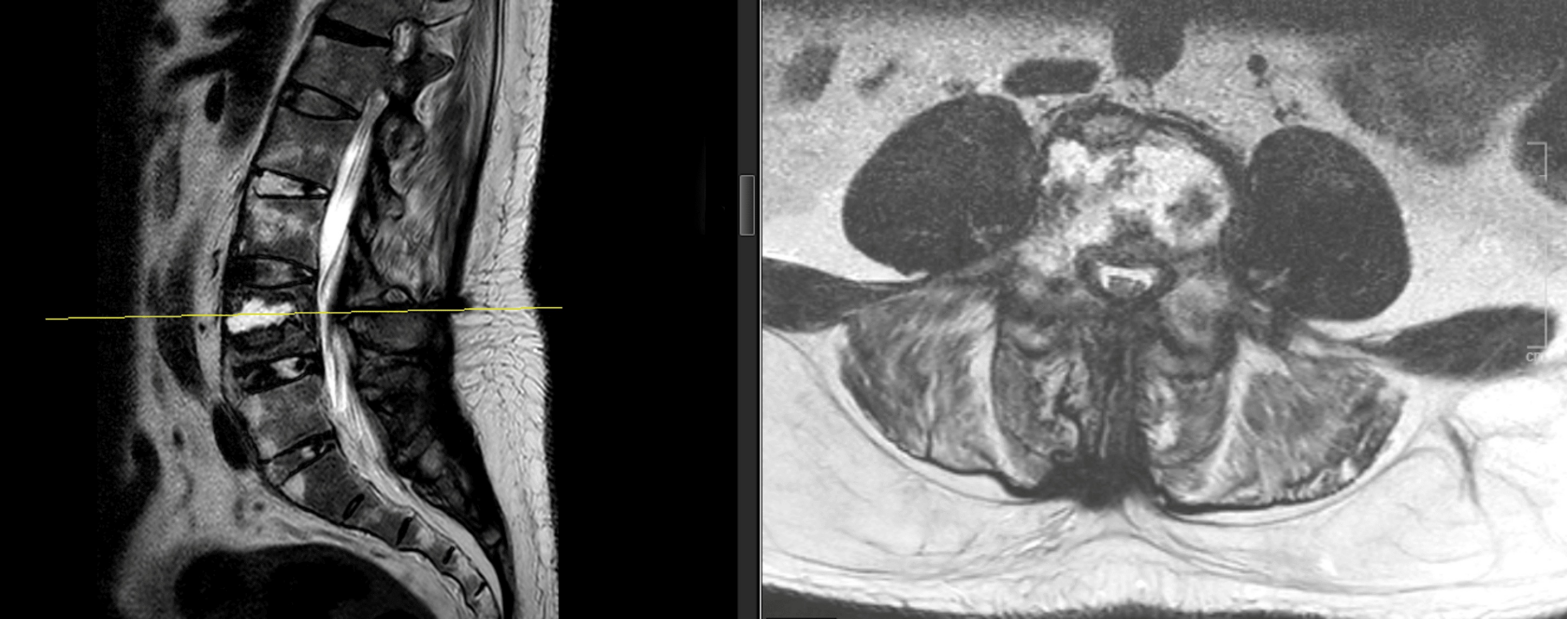
T2-weighted image shows L4 vertebra chance fracture causing the abutment of bilateral L4 traversing nerve roots and possibly the impingement of left L4 exiting nerve root

On magnetic resonance imaging (MRI), T2 short tau inversion recovery (STIR) sequence disclosed high signal intensity from the body and neural arch, most likely due to posttraumatic edema consistent with fracture. A cervical MRI shows a fracture over the C6 body with evidence of spinal stenosis (Figure 3), while a lumbosacral MRI revealed a chance fracture of L4 (Figure 4).

**Figure 5:**
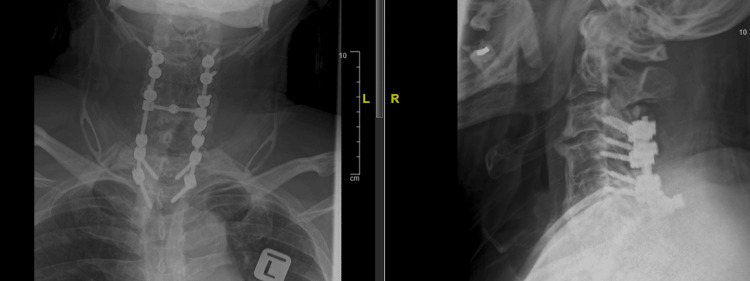
Anteroposterior and lateral cervical radiograph post instrumentation shows instrumentation three levels above and below the C6 vertebrae

**Figure 6:**
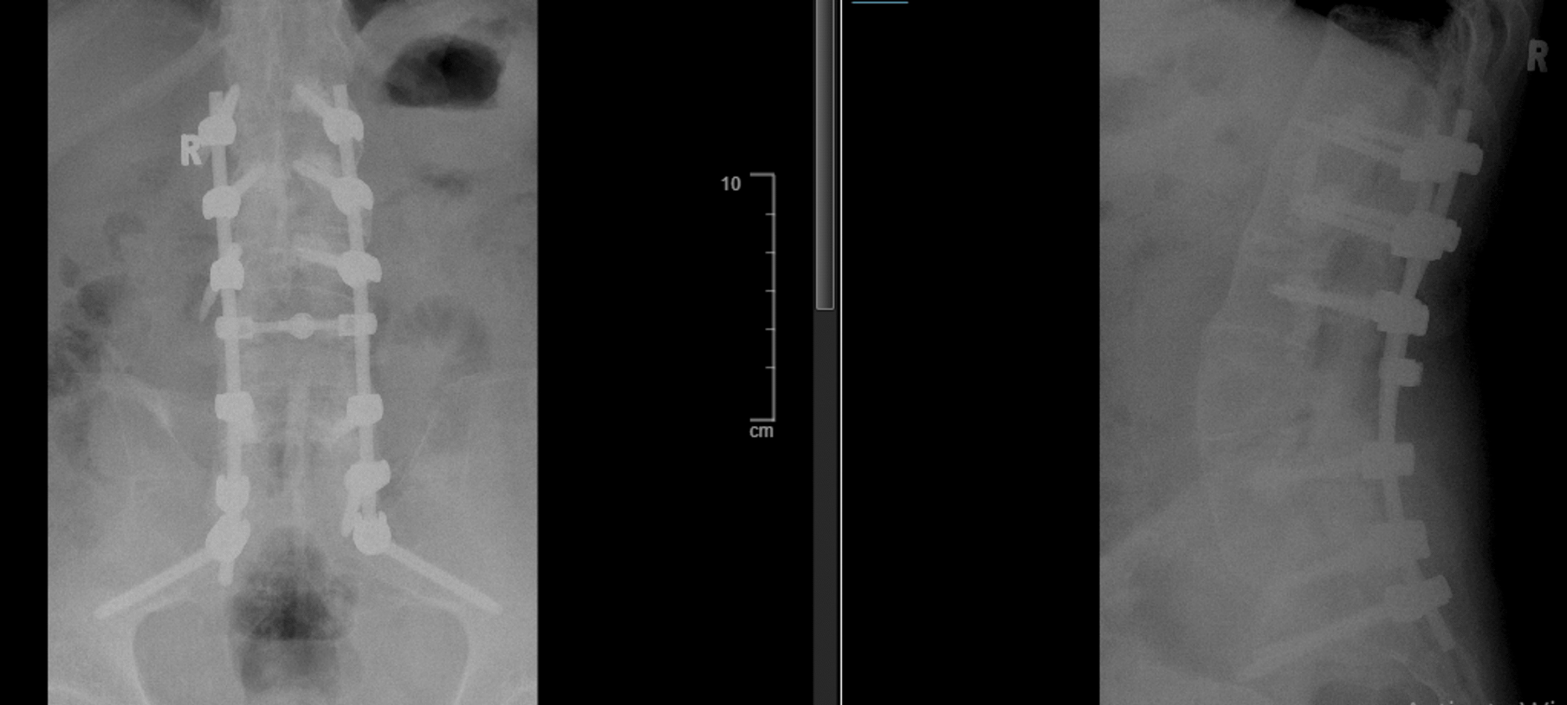
Anteroposterior and lateral view of a lumbosacral X-ray postoperatively: PSIF from L1 to S2 with cement augmentation PSIF: posterior spinal instrumentation and fusion

This fracture in a patient with AS involved all three spinal columns and was therefore considered unstable. He underwent posterior spinal instrumentation and fusion (PSIF) from C3 to T2 (Figure 5) and PSIF from L1 to S2 (Figure 6) with bone cement augmentation and L4/L5 decompression laminectomy. The procedure was done under fluoroscopy guidance as the areas of fusion were blurred due to the AS. The decision to fix with instrumentation extending over three vertebrae above and three below the fracture level was made due to compact cortical bone contrasting with demineralization within the vertebral bodies that requires multilevel anchoring. This ensures favorable stress distribution on the implants, providing additional stability along the sagittal plane.

Postoperatively, the patient was able to ambulate with a wheelchair and responded well following the rehabilitation regimen. The neurological status remains the same as preoperative.

## Discussion

The simultaneous occurrence of cervical and lumbar vertebral fractures is an exceedingly rare and unusual clinical presentation in patients with AS. The cervical spine is typically more mobile than the lumbar spine and, therefore, more vulnerable to trauma. In AS, the loss of flexibility can predispose the cervical spine to fractures with associated dislocations. The inability of the spinal column to absorb forces normally transmitted through the flexible vertebral joints may lead to fractures-dislocations [3]. The lumbar spine, although more rigid in AS, is still susceptible to fractures, particularly when subjected to significant trauma. The loss of normal curvature and biomechanical function of the lumbar spine, in addition to its relatively greater immobility, can result in a more rigid fracture pattern. The simultaneous occurrence of lumbar and cervical injuries is often indicative of an event involving considerable forces or trauma, such as motor vehicle accidents or falls from significant heights.

Diagnosing fractures in patients with AS can be challenging due to the presence of vertebral fusion and altered bone architecture. The usual radiographic signs of fracture, such as displacement, may be masked by the lack of joint mobility and ossification. CT scans and MRI are invaluable in providing a more detailed assessment of fracture-dislocation patterns, soft tissue involvement, and potential spinal cord injury [2].

The prognosis of AS patients with simultaneous cervical and lumbar fractures is contingent upon the severity of neurological impairment, the extent of the fractures, and the success of the surgical intervention. The mismatch between rigid spinal implants and osteopenic bone can lead to catastrophic implant failure due to the high stresses placed on the bone-implant interface. To address this issue, techniques such as cement augmentation of pedicle screws have been developed to enhance fixation [4]. By reinforcing spinal hardware with cement, the strength of the bone-implant interface and the pullout resistance of the screws can be improved, potentially leading to better long-term outcomes and increased implant longevity.

Posterior long-segment (LS) fixation, short-segment (SS) fixation, and short-segment fixation with intermediate screws (SI) have shown good outcomes for the treatment of thoracolumbar burst fractures [5]. Posterior short-segment instrumentation (SI) preserves more spinal motion compared to long-segment (LS) instrumentation. Additionally, it offers more favorable biomechanical properties in terms of sagittal balance. Among the three constructs, SI generates the least stress around the pedicle screws, which may help reduce the risk of complications such as implant failure [6].

This patient underwent posterior spinal instrumentation and fusion (PSIF) from L1 to S2 and C3 to T2, with bone cement augmentation, as well as L4/L5 decompression laminectomy.

Combined with a proper rehab regimen, he was able to ambulate by himself after 3/12 postoperatively.

## Conclusions

The occurrence of simultaneous cervical and lumbar vertebral fractures-dislocations in a patient with ankylosing spondylitis is a rare and challenging clinical situation. The pathological changes associated with AS make the spine more susceptible to fractures, even with low-energy trauma, and this case highlights the importance of early and accurate diagnosis. Management involves early stabilization, potentially complex surgical intervention, and rehabilitation to optimize functional recovery. Understanding the unique presentation and challenges associated with AS patients is vital for improving outcomes in such rare cases.
